# Supplementary material for: Resolving the full spectrum of human genome variation using Linked-Reads
Source: Genome Res. 2019 Apr;29(4):635–45. doi: 10.1101/gr.234443.118 (PMC6442396; doi:10.1101/gr.234443.118)
Supplement: Supplemental Material [file supp_gr.234443.118_Supplemental_longranger-2.2.zip › longranger-2.2.tar.bz2!/lr2.2-public/lib/stan/cmdstan/stan/doc/api/html/classstan_1_1io_1_1reader-members.html]

Stan: Member List


|  |  |
| --- | --- |
|  | Stan  2.5.0  probability, sampling & optimization |


- Main Page
- Related Pages
- Namespaces
- Classes
- Files

- Class List
- Class Index
- Class Hierarchy
- Class Members

All Classes Namespaces Files Functions Variables Typedefs Enumerations Enumerator Friends Macros Pages

- stan
- io
- reader

stan::io::reader< T > Member List

This is the complete list of members for stan::io::reader< T >, including all inherited members.

|  |  |  |
| --- | --- | --- |
| available() | stan::io::reader< T > | inline |
| available\_i() | stan::io::reader< T > | inline |
| cholesky\_corr(size\_t K) | stan::io::reader< T > | inline |
| cholesky\_corr\_constrain(size\_t K) | stan::io::reader< T > | inline |
| cholesky\_corr\_constrain(size\_t K, T &lp) | stan::io::reader< T > | inline |
| cholesky\_factor(size\_t M, size\_t N) | stan::io::reader< T > | inline |
| cholesky\_factor\_constrain(size\_t M, size\_t N) | stan::io::reader< T > | inline |
| cholesky\_factor\_constrain(size\_t M, size\_t N, T &lp) | stan::io::reader< T > | inline |
| corr() | stan::io::reader< T > | inline |
| corr\_constrain() | stan::io::reader< T > | inline |
| corr\_constrain(T &lp) | stan::io::reader< T > | inline |
| corr\_matrix(size\_t k) | stan::io::reader< T > | inline |
| corr\_matrix\_constrain(size\_t k) | stan::io::reader< T > | inline |
| corr\_matrix\_constrain(size\_t k, T &lp) | stan::io::reader< T > | inline |
| cov\_matrix(size\_t k) | stan::io::reader< T > | inline |
| cov\_matrix\_constrain(size\_t k) | stan::io::reader< T > | inline |
| cov\_matrix\_constrain(size\_t k, T &lp) | stan::io::reader< T > | inline |
| integer() | stan::io::reader< T > | inline |
| integer\_constrain() | stan::io::reader< T > | inline |
| integer\_constrain(T &) | stan::io::reader< T > | inline |
| integer\_lb(int lb) | stan::io::reader< T > | inline |
| integer\_lb\_constrain(int lb) | stan::io::reader< T > | inline |
| integer\_lb\_constrain(int lb, T &) | stan::io::reader< T > | inline |
| integer\_lub(int lb, int ub) | stan::io::reader< T > | inline |
| integer\_lub\_constrain(int lb, int ub) | stan::io::reader< T > | inline |
| integer\_lub\_constrain(int lb, int ub, T &) | stan::io::reader< T > | inline |
| integer\_ub(int ub) | stan::io::reader< T > | inline |
| integer\_ub\_constrain(int ub) | stan::io::reader< T > | inline |
| integer\_ub\_constrain(int ub, T &) | stan::io::reader< T > | inline |
| map\_matrix\_t typedef | stan::io::reader< T > |  |
| map\_row\_vector\_t typedef | stan::io::reader< T > |  |
| map\_vector\_t typedef | stan::io::reader< T > |  |
| matrix(size\_t m, size\_t n) | stan::io::reader< T > | inline |
| matrix\_constrain(size\_t m, size\_t n) | stan::io::reader< T > | inline |
| matrix\_constrain(size\_t m, size\_t n, T &) | stan::io::reader< T > | inline |
| matrix\_lb(const TL lb, size\_t m, size\_t n) | stan::io::reader< T > | inline |
| matrix\_lb\_constrain(const TL lb, size\_t m, size\_t n) | stan::io::reader< T > | inline |
| matrix\_lb\_constrain(const TL lb, size\_t m, size\_t n, T &lp) | stan::io::reader< T > | inline |
| matrix\_lub(const TL lb, const TU ub, size\_t m, size\_t n) | stan::io::reader< T > | inline |
| matrix\_lub\_constrain(const TL lb, const TU ub, size\_t m, size\_t n) | stan::io::reader< T > | inline |
| matrix\_lub\_constrain(const TL lb, const TU ub, size\_t m, size\_t n, T &lp) | stan::io::reader< T > | inline |
| matrix\_t typedef | stan::io::reader< T > |  |
| matrix\_ub(const TU ub, size\_t m, size\_t n) | stan::io::reader< T > | inline |
| matrix\_ub\_constrain(const TU ub, size\_t m, size\_t n) | stan::io::reader< T > | inline |
| matrix\_ub\_constrain(const TU ub, size\_t m, size\_t n, T &lp) | stan::io::reader< T > | inline |
| ordered(size\_t k) | stan::io::reader< T > | inline |
| ordered\_constrain(size\_t k) | stan::io::reader< T > | inline |
| ordered\_constrain(size\_t k, T &lp) | stan::io::reader< T > | inline |
| positive\_ordered(size\_t k) | stan::io::reader< T > | inline |
| positive\_ordered\_constrain(size\_t k) | stan::io::reader< T > | inline |
| positive\_ordered\_constrain(size\_t k, T &lp) | stan::io::reader< T > | inline |
| prob() | stan::io::reader< T > | inline |
| prob\_constrain() | stan::io::reader< T > | inline |
| prob\_constrain(T &lp) | stan::io::reader< T > | inline |
| reader(std::vector< T > &data\_r, std::vector< int > &data\_i) | stan::io::reader< T > | inline |
| row\_vector(size\_t m) | stan::io::reader< T > | inline |
| row\_vector\_constrain(size\_t m) | stan::io::reader< T > | inline |
| row\_vector\_constrain(size\_t m, T &) | stan::io::reader< T > | inline |
| row\_vector\_lb(const TL lb, size\_t m) | stan::io::reader< T > | inline |
| row\_vector\_lb\_constrain(const TL lb, size\_t m) | stan::io::reader< T > | inline |
| row\_vector\_lb\_constrain(const TL lb, size\_t m, T &lp) | stan::io::reader< T > | inline |
| row\_vector\_lub(const TL lb, const TU ub, size\_t m) | stan::io::reader< T > | inline |
| row\_vector\_lub\_constrain(const TL lb, const TU ub, size\_t m) | stan::io::reader< T > | inline |
| row\_vector\_lub\_constrain(const TL lb, const TU ub, size\_t m, T &lp) | stan::io::reader< T > | inline |
| row\_vector\_t typedef | stan::io::reader< T > |  |
| row\_vector\_ub(const TU ub, size\_t m) | stan::io::reader< T > | inline |
| row\_vector\_ub\_constrain(const TU ub, size\_t m) | stan::io::reader< T > | inline |
| row\_vector\_ub\_constrain(const TU ub, size\_t m, T &lp) | stan::io::reader< T > | inline |
| scalar() | stan::io::reader< T > | inline |
| scalar\_constrain() | stan::io::reader< T > | inline |
| scalar\_constrain(T &) | stan::io::reader< T > | inline |
| scalar\_lb(const TL lb) | stan::io::reader< T > | inline |
| scalar\_lb\_constrain(const TL lb) | stan::io::reader< T > | inline |
| scalar\_lb\_constrain(const TL lb, T &lp) | stan::io::reader< T > | inline |
| scalar\_lub(const TL lb, const TU ub) | stan::io::reader< T > | inline |
| scalar\_lub\_constrain(const TL lb, const TU ub) | stan::io::reader< T > | inline |
| scalar\_lub\_constrain(TL lb, TU ub, T &lp) | stan::io::reader< T > | inline |
| scalar\_pos() | stan::io::reader< T > | inline |
| scalar\_pos\_constrain() | stan::io::reader< T > | inline |
| scalar\_pos\_constrain(T &lp) | stan::io::reader< T > | inline |
| scalar\_ub(TU ub) | stan::io::reader< T > | inline |
| scalar\_ub\_constrain(const TU ub) | stan::io::reader< T > | inline |
| scalar\_ub\_constrain(const TU ub, T &lp) | stan::io::reader< T > | inline |
| simplex(size\_t k) | stan::io::reader< T > | inline |
| simplex\_constrain(size\_t k) | stan::io::reader< T > | inline |
| simplex\_constrain(size\_t k, T &lp) | stan::io::reader< T > | inline |
| std\_vector(size\_t m) | stan::io::reader< T > | inline |
| unit\_vector(size\_t k) | stan::io::reader< T > | inline |
| unit\_vector\_constrain(size\_t k) | stan::io::reader< T > | inline |
| unit\_vector\_constrain(size\_t k, T &lp) | stan::io::reader< T > | inline |
| vector(size\_t m) | stan::io::reader< T > | inline |
| vector\_constrain(size\_t m) | stan::io::reader< T > | inline |
| vector\_constrain(size\_t m, T &) | stan::io::reader< T > | inline |
| vector\_lb(const TL lb, size\_t m) | stan::io::reader< T > | inline |
| vector\_lb\_constrain(const TL lb, size\_t m) | stan::io::reader< T > | inline |
| vector\_lb\_constrain(const TL lb, size\_t m, T &lp) | stan::io::reader< T > | inline |
| vector\_lub(const TL lb, const TU ub, size\_t m) | stan::io::reader< T > | inline |
| vector\_lub\_constrain(const TL lb, const TU ub, size\_t m) | stan::io::reader< T > | inline |
| vector\_lub\_constrain(const TL lb, const TU ub, size\_t m, T &lp) | stan::io::reader< T > | inline |
| vector\_t typedef | stan::io::reader< T > |  |
| vector\_ub(const TU ub, size\_t m) | stan::io::reader< T > | inline |
| vector\_ub\_constrain(const TU ub, size\_t m) | stan::io::reader< T > | inline |
| vector\_ub\_constrain(const TU ub, size\_t m, T &lp) | stan::io::reader< T > | inline |
| ~reader() | stan::io::reader< T > | inline |


---

[ Stan Home Page ]

*© 2011–2014,
Stan Development Team.*
